# Increasing Female Representation Among Presenters at the Arthroscopy Association of North America Annual Meetings From 2015 to 2023

**DOI:** 10.7759/cureus.90105

**Published:** 2025-08-14

**Authors:** Edward J Modica, Allison Lewis, Kathryn Persin, Joshua Gruber, Brandon Klein, Randy Cohn, Nicholas A Sgaglione

**Affiliations:** 1 Orthopaedic Surgery, Northwell Health, Huntington, USA; 2 Orthopaedics, Northwell Health, Huntington, USA; 3 Osteopathic Medicine, Philadelphia College of Osteopathic Medicine, Philadelphia, USA; 4 Orthopaedic Surgery, University of Pittsburgh Medical Center Pinnacle, Harrisburg, USA; 5 Orthopaedic Surgery, Northwell Health, Garden City, USA; 6 Orthopaedic Surgery, Northwell Health, Long Island Jewish Medical Center, New Hyde Park, USA

**Keywords:** annual meeting, female orthopedic surgeon, gender representation, orthopedic sports medicine, orthopedic sugery

## Abstract

Purpose

The purpose of this study was to analyze the representation of female presenters across various roles at the Arthroscopy Association of North America (AANA) annual meetings held between 2015 and 2023.

Methods

Conference programs from the 2015-2023 AANA annual meetings were reviewed. Meeting presenters were categorized by presenter gender (male and female) and role type (academic or invited). Academic roles were subdivided into paper presentation moderators and paper presentations. Invited roles were further subdivided into symposium moderators, symposium lecturers, panel moderators, and panel members. The content presented was categorized into eight areas: shoulder, elbow, hand, hip, knee, foot and ankle, biologics, and presentations encompassing multiple categories. Female representation among roles and content categories was analyzed using means, proportions, and statistical tests such as two-tailed z-tests and the Pearson correlation coefficient. A p-value <0.05 was considered significant. All statistics were performed on Microsoft Excel (Microsoft Corporation, Redmond, WA).

Results

There were 1562 total presenters from 2015 to 2023. Overall, there was an average of 22 female presenters each year, with the highest number of female presenters in 2022 (n = 49). The proportion of female presenters over the study period increased (p < 0.01). While there was no trend in the proportion of female presenters in academic roles (r = 0.20, p = 0.66), there was an increase in the proportion of female presenters in invited roles (r = 0.99, p < 0.01). Among academic roles, there was an increase in female representation among paper presentation moderators (r = 0.93, p < 0.01) and panel members (r = 0.97, p < 0.01). In addition, there was increasing female representation among presentations on the knee (r = 0.83, p = 0.02), biologics (r = 0.83, p = 0.04), and topics encompassing multiple categories (r = 0.89, p < 0.01).

Conclusion

This study highlights a significant increase in female representation among presenters at the AANA annual meetings from 2015 to 2023, particularly in invited roles and certain content categories like knee, biologics, and multi-category presentations. Collaborations with the Ruth Jackson Orthopaedic Society (RJOS) and the FORUM have had a positive impact on female representation. The increasing trend suggests that ongoing diversity initiatives by AANA, such as the Diversity & Inclusion Task Force, may be contributing to this progress.

## Introduction

With an increasing awareness of gender disparities across healthcare, academic institutions and national organizations have sought to improve workplace diversity within their programs and the broader medical profession [1-5]. An increase in diversity among physician teams has been linked to higher productivity, improved satisfaction among patients, and better overall patient outcomes [6-9]. Within orthopedic surgery, there has been an increase in the proportion of female orthopedic surgeons at both the attending (5.3% to 8.0%) and various in-training (12.1% to 17%) levels from 2018 to 2023 [10]. Although the specialty remains male-dominated, recent data continue to reflect a gradual but steady rise in the percentage of female applicants applying for and matching into orthopedic surgery residencies [11].

The recent recognition of barriers to gender equality within orthopedic surgery has led to targeted initiatives to eliminate gender discrepancies. In 2018, the American Academy of Orthopaedic Surgeons (AAOS) launched a five-year strategic plan to advance initiatives focused on “leader recruitment, selection, retention, culture, and diversity” [12]. As a result, the number of female members serving on the AAOS board of directors or other AAOS committees increased substantially to 17% from 2018 to 2023 [12]. Despite these initiatives and nationally driven directives guiding gender diversity, female orthopedic surgeons continue to be underrepresented within specific areas of orthopedic surgery, including senior authorship in major orthopedic journals, service as academic faculty, leadership roles, and representation within orthopedic societies [13-16]. Conferences held by professional societies, such as the Arthroscopy Association of North America (AANA), a leading society in the field of orthopedic sports medicine, offer a valuable opportunity for professional development. This is achieved through the presentation of original work or speaking on one’s contributions to the field at these annual meetings. There remains a concern regarding gender equality across various speaking roles at these meetings. In a cross-sectional study of 17 national orthopedic societies, Gerull et al. reported that female speakers from societies without stated diversity efforts were poorly represented at conferences and annual meetings [17]. Additionally, they were more likely to speak on non-technical topics such as work-life balance, social media, or education [17]. Assigning female orthopedic surgeons to predominantly non-academic roles may hinder their opportunities to enhance their academic standing. For academic orthopedic surgeons, career advancement may depend on research productivity, national and international reputation, and academic rank [18].

As a leading society in the field of orthopedic sports medicine, the AANA plays a significant role in shaping the specialty. Its "Diversity & Inclusion Statement" affirms a commitment to supporting a diverse and inclusive membership through programming and leadership that fosters understanding and respect for all individuals. They specifically note that diversity contributes to innovation and success in minimally invasive orthopedic surgery [19]. However, there is limited literature evaluating gender diversity at the AANA annual meetings.

The purpose of this study was to analyze the representation of female presenters across various roles at the AANA annual meetings held between 2015 and 2023. The authors sought to answer the following questions: (1) What proportion of presenters are female among specific roles and content categories? (2) Has there been a change in the proportion of presenters who are female, and are there specific roles that have experienced a change across the study period? Given the AAOS initiative to increase diversity, we hypothesize that female representation has increased at the AANA annual meetings.

## Materials and methods

The 2015-2023 AANA annual meeting conference programs were obtained as portable document format (PDF) files through contact with AANA via email. The 2021 meeting was excluded as it was a combined meeting with the American Orthopaedic Society for Sports Medicine (AOSSM), and there was no meeting held in 2020 due to the COVID-19 pandemic. Approval from the Institutional Review Board (IRB) was not required as this study did not include human subjects.

Each conference program was independently screened by two reviewers for all presenters with an MD (doctor of medicine) or DO (doctor of osteopathic medicine) degree designation. Industry-sponsored presentations, resident-centered education, instructional courses, and business content were excluded to focus on academic lecture-based content only. Male and female presenters were determined based on publicly available information through the internet, via professional websites, and listed biographies. The presenter’s name, online photo, and pronouns used in their biographies were all used to determine gender. Each presenter was assigned to either the academic or invited role type. Academic roles were determined to be any role that included presenting or moderating academic papers. Academic roles included paper presentations and paper presentation moderators. For paper presentations, presenters were assumed to be the person whose name was bolded on the program. If no name was bolded, it was assumed that the first name listed was the presenter. Invited roles were determined to be any role that did not include presenting or moderating academic paper presentations. Invited roles were subdivided into symposium moderators, symposium lecturers, panel moderators, and panel members [17,20]. The content area presented was categorized into one of eight topics: shoulder, elbow, hand, hip, knee, foot and ankle, biologics, and presentations encompassing multiple categories. Any disputes in categorization were settled at the discretion of a third reviewer.

The number of female presenters in each role type, specific role, and content category was analyzed using means and proportions. The proportion of females in academic and invited roles was compared using a two-tailed Z-test for proportions. The trends in female representation across role types, specific roles, and content categories were analyzed using the Pearson correlation coefficient (r). P-values less than 0.05 were considered significant. All statistics were performed on Microsoft Excel (Microsoft Corporation, Redmond, WA).

## Results

**Figure 1:**
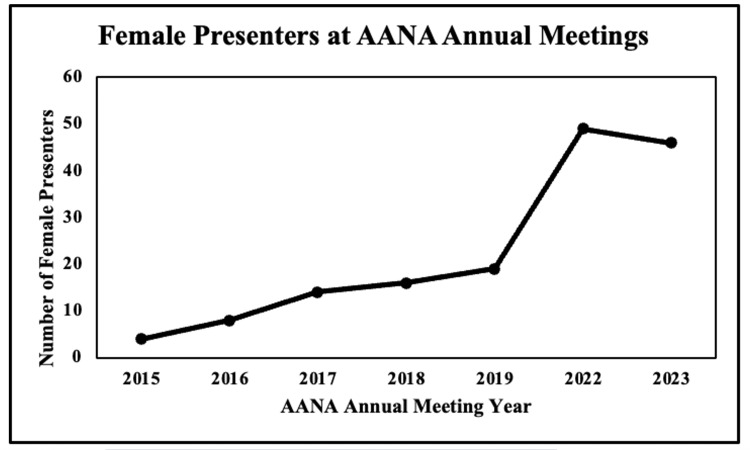
Female presenters at the AANA annual meetings. This graph represents the total number of female presenters at the Arthroscopy Association of North America (AANA) annual meetings from 2015 to 2023.

There were a total of 1562 presentations at the 2015-2023 AANA annual meetings that met the inclusion criteria. There were 156 (10.0%) presentations given by female presenters, with an average of 22 female presenters per year. The highest number of female presenters occurred in 2022 (n = 49). Over the study period, there was an increase in the proportion of presentations given by female surgeons (r = 0.94, p < 0.01) (Figure 1).

**Figure 2:**
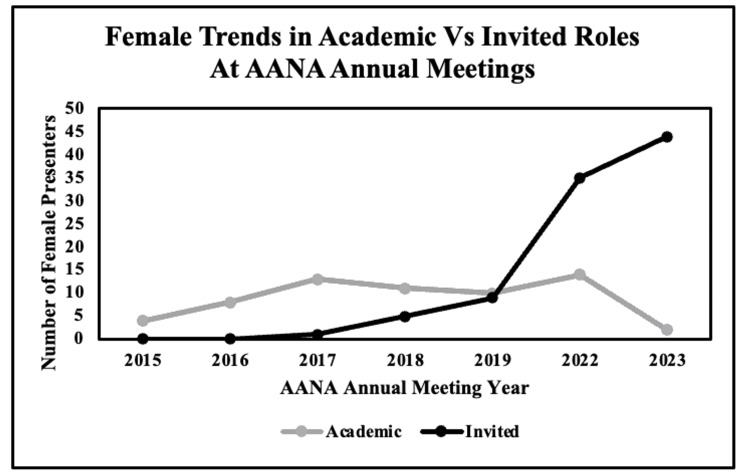
Female trends in academic vs. invited roles at the AANA annual meetings. This graph represents the total number of female presenters selected for academic roles and invited roles to the Arthroscopy Association of North America (AANA) annual meeting from 2015 to 2023.

There were 580 presentations in academic roles and 982 in invited roles. Of the academic roles, 11% (62/580) were female. There was no trend in the proportion of female presenters in academic roles over our study period (r = 0.20, p = 0.66). Females represented 10% (94/888) of invited roles, with an increase in the proportion of female presenters in invited roles over the study period (r = 0.99, p < 0.01). There was no difference in the proportion of females in academic vs. invited roles (p = 0.52) (Figure 2).

**Figure 3:**
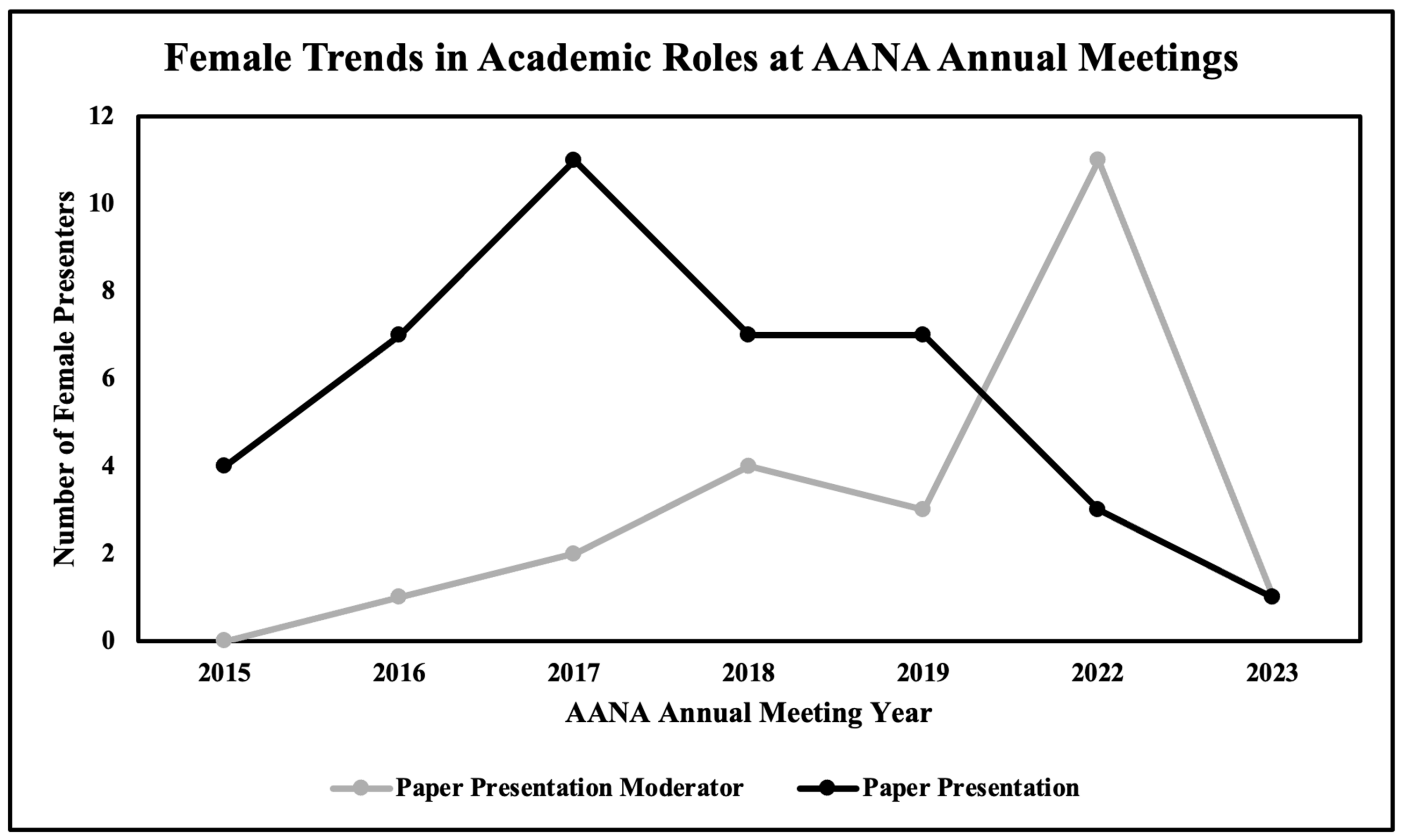
Female trends in academic roles at the AANA annual meetings. This graph represents the total number of female presenters selected for poster presentations and paper presentations at the Arthroscopy Association of North America (AANA) annual meetings from 2015 to 2023.

**Figure 4:**
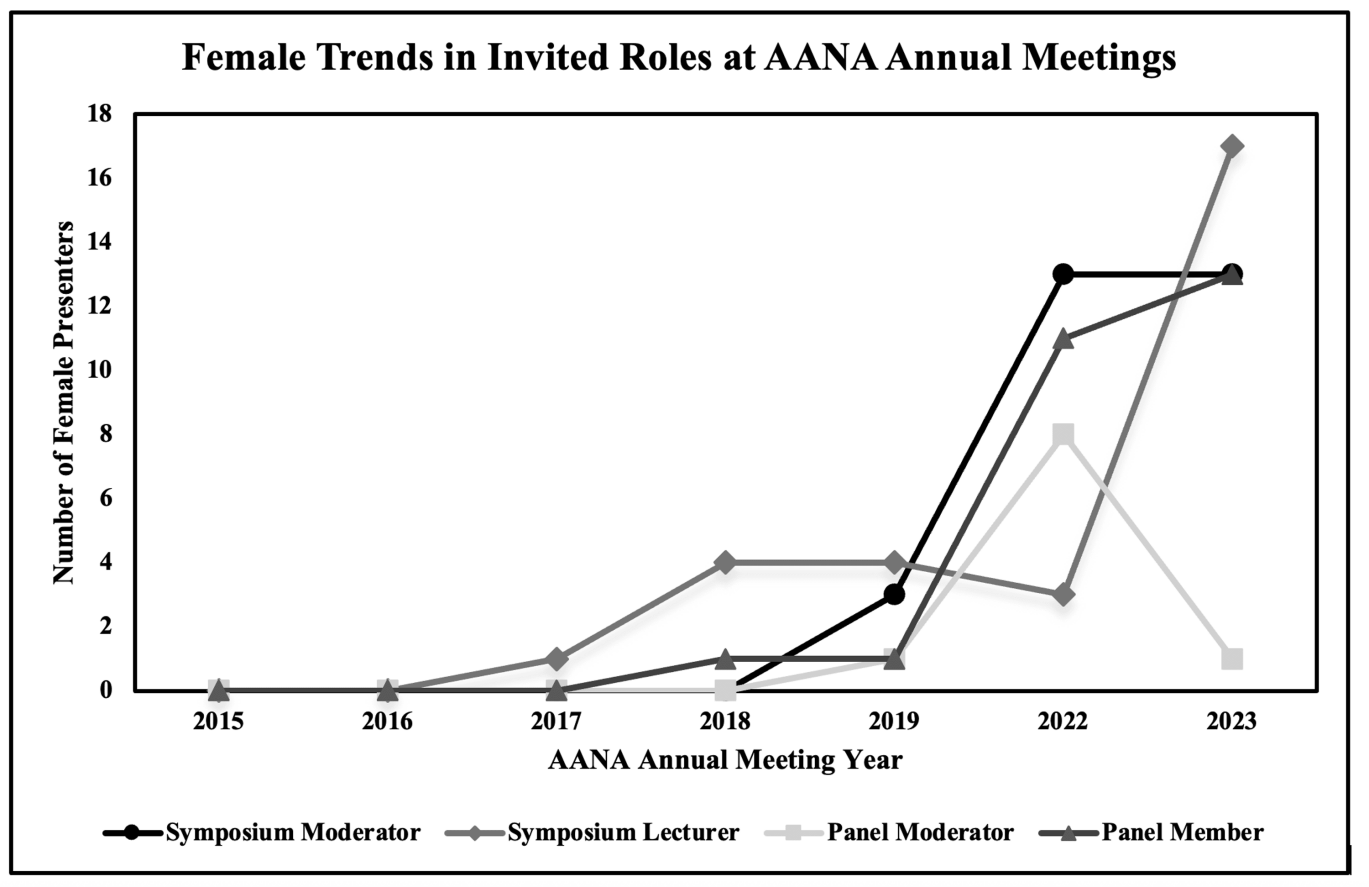
Female trends in invited roles at the AANA annual meetings. This graph represents the total number of female presenters selected as symposium moderators, symposium lecturers, panel moderators, and panel members at the Arthroscopy Association of North America (AANA) annual meetings from 2015 to 2023.

Females comprised 25% (29/115) of symposium moderators, 7% (29/414) of symposium lecturers, 14% (22/153) of paper presentation moderators, 9% (40/427) of paper presentations, 10% (10/97) of panel moderators, and 7% (26/356) of panel members. The proportion of female paper presentation moderators (r = 0.93, p < 0.01) and panel members (r = 0.97, p < 0.01) increased over the study period. There were no trends in female representation identified among any other assigned roles (Figures 3, 4).

In evaluation of presenters by content category, females comprised 6% (33/548) of shoulder presentations, 17% (92/541) of knee presentations, 6% (12/202) of hip presentations, 3% (3/88) of foot and ankle presentations, 2% (1/66) of elbow presentations, 11% (6/56) of biologics presentations, 0% (0/1) of hand presentations, and 15% (9/60) of presentations encompassing multiple categories. The proportion of females presenting on the knee (r = 0.83, p = 0.02), biologics (r = 0.83, p = 0.04), and topics encompassing multiple categories (r = 0.89, p < 0.01) increased over the study period. There were no other trends in female representation in any other content category.

## Discussion

Previous literature has illustrated that societies with defined diversity efforts demonstrate a larger percentage of female speakers than those without [17]. AANA’s diversity and inclusion statement, along with the Diversity & Inclusion Task Force initiatives, supports this sentiment [19]. During the nine-year study period, this study found an increase in the proportion of presenters who were female. There was also a statistically significant increase in the proportion of female speakers in invited roles during the study period. These increases in female representation could be partially attributed to AANA’s multiple diversity initiatives, but the direct impact on measurable improvements in gender diversity has not been established.

The increase in female presenters in 2022 and 2023 could also be attributed to the AANA’s collaboration with the Ruth Jackson Orthopaedic Society (RJOS) and the FORUM. Both professional societies seek to promote the development of women in orthopedics [21,22]. Future collaboration with these societies will only improve opportunities for female orthopedic surgeons and help increase representation across annual meetings.

The content categories with the highest proportion of female presenters (knee, mixed content, and biologics) showed an increasing trend in female representation over the study period. This pattern may reflect broader trends within orthopedic sports medicine. Knee-related procedures represent a major focus of the specialty, accounting for up to 60% of all sports-related surgeries, while biologics are a topic of much scrutiny due to their exponential growth over the last decade, theoretical healing potential, and current lack of high-quality evidence [23,24]. Therefore, these findings may suggest that female representation is increasing in categories that are central to clinical practice and at the forefront of emerging research and debate.

Overall, there was a higher proportion of presentations at the AANA annual meetings given by females (10%) than the overall proportion of practicing orthopedic surgeons who are female, which was reported as 6% in 2020 [25]. This coincides with previous literature investigating gender trends of speakers at national orthopedic conference meetings [26].

Annual society meetings serve as crucial opportunities for gaining visibility, forming professional networks, and establishing a national profile [25]. For orthopedic societies committed to gender equality initiatives, conducting a thorough examination of presenter demographics at annual meetings could offer valuable insights into this important issue. Prior studies have highlighted gender disparities in the assignment of roles at orthopedic society gatherings [17]. A significant strength of this research is that the study includes data from seven meetings spanning over nine years, allowing it to identify the current trend and chart progress over time.

Limitations

This study was not without limitations. First, the project faced limitations partly due to the non-scientific nature of the presenter selection process, which inherently skews measurement efforts. It can be assumed that not every individual invited to speak accepts the invitation, which would force the selection committee to move to the next candidate. This adds a measure of unpredictability to the selection process. Additionally, there are likely many nuances in the synthesis of each program that could not be accounted for. Second, the determination of the presenter’s gender was based on publicly available information through the internet, without a method of confirming the gender identity of the presenter. Each presenter’s gender was determined by an agreement with two authors. Additionally, a binary gender classification was used and thus does not capture the full spectrum of gender. Third, role types and content categories were determined by the authors, and presentations were subjectively selected into these categories. However, a third reviewer was utilized to settle any disagreements over role type and content categorization. Furthermore, this study was not able to evaluate year-by-year changes due to the absence of a conference in 2020 and the combined AANA/AOSSM conference in 2021. Other limitations included the small number of hand presentations and the exclusion of non-physician speakers from our analysis. Finally, the authors recognize that gender identity is not the only metric used when selecting presenters, and conference planning committees devote substantial time to determining experts for their selection for invited roles.

## Conclusions

This study highlights a significant increase in female representation among presenters at the AANA annual meetings from 2015 to 2023, particularly in invited roles and certain content categories like knee, biologics, and multi-category presentations. Collaborations with the RJOS and the FORUM have had a positive impact on female representation. The increasing trend suggests that ongoing diversity initiatives by AANA, such as the Diversity & Inclusion Task Force, may be contributing to this progress.
